# Performance Comparison Between Plasma and Stool Methylated *SEPT9* Tests for Detecting Colorectal Cancer

**DOI:** 10.3389/fgene.2020.00324

**Published:** 2020-04-16

**Authors:** Yi Liu, Guodong Zhao, Jin Miao, Hui Li, Yong Ma, Xiaoyu Liu, Shiming Li, Yun Zhu, Shangmin Xiong, Minxue Zheng, Sujuan Fei

**Affiliations:** ^1^Department of Gastroenterology, Affiliated Hospital of Xuzhou Medical University, Xuzhou, China; ^2^Institute of Digestive Diseases, Xuzhou Medical University, Xuzhou, China; ^3^Zhejiang University Kunshan Biotechnology Laboratory, Zhejiang University Kunshan Innovation Institute, Kunshan, China; ^4^State Key Laboratory of Bioelectronics, School of Biological Science and Medical Engineering, Southeast University, Nanjing, China; ^5^Suzhou VersaBio Technologies Co., Ltd., Kunshan, China; ^6^Suzhou Institute of Biomedical Engineering and Technology, Chinese Academy of Sciences, Suzhou, China

**Keywords:** methylated *SEPT9*, plasma, stool, colorectal cancer, screening

## Abstract

Colorectal cancer (CRC) is the most common type of malignancies of the gastrointestinal tract worldwide. Plasma methylated *SEPT9* test has been used clinically for CRC screening for several years, but the study about the performance comparison between plasma and stool has rarely been reported. In this study, 124 plasma samples, 100 stool samples, and 60 sets of plasma and paired stool samples were collected and tested by a methylated *SEPT9* test in three PCR replicates. The results indicated methylated *SEPT9* levels in stool samples were significant higher than those in plasma samples (*p* < 0.0001). When a plasma sample was called positive if 1 out of 3 PCR replicates was positive and a stool sample was called positive if 3 out of 3 PCR replicates were positive with a mean Cp value of less than 40.0, stool methylated *SEPT9* test achieved similar sensitivity (83.3% vs 85.6%) and specificity (92.1% vs 90.1%) to those by plasma methylated *SEPT9* test, and the overall concordance rate is 78.3%. However, stool methylated *SEPT9* test showed 35.9 and 7.9% improvement in detecting advanced adenomas (AA) and stage I–II CRC in comparison to plasma methylated *SEPT9* test. The AUC for plasma methylated *SEPT9* and stool methylated *SEPT9* in detecting CRC were 0.885 (95% CI: 0.832–0.938) and 0.935 (95% CI: 0.895–0.975), respectively. In conclusion, stool methylated *SEPT9* test showed higher sensitivities for detection AA and early stage CRC compared with plasma methylated SEPT9 test, and stool methylated *SEPT9* test may be a more suitable tool for early stage CRC screening.

## Introduction

Colorectal cancer (CRC) is the most common malignancy of gastrointestinal tract and the third most common cancer types all over the world (Schreuders et al., 2015). It is also the fifth most common cancerin China (Chen et al., 2016). With the development of Chinese economy and the increase of residents’ income, the lifestyle and dietary habits of Chinese population is gradually westernizing, and the incidence of CRC has seen steady increase in recent years, especially for urban population. In the past few years, the 5 year relative survival rate of CRC patients in China has increased from 47.2 to 56.9%, but it is still more than 8% lower than that of the developed countries (Siegel et al., 2017; Zeng et al., 2018). Based on epidemiologic studies in United States and Japan, long-standing CRC screening and early detection programs had a significant role in reducing morbidity and mortality (Bray et al., 2018).

The recommended options for CRC screening by the recently updated guideline for average-risk adults from the American Cancer Society (ACS) include fecal immunochemical test (FIT) annually, guaiac-based fecal occult blood test (gFOBT) annually, multi-target stool DNA test every 3 years, colonoscopy every 10 years, computed tomography colonography every 5 years, and flexible sigmoidoscopy every 5 years (Wolf et al., 2018). However, the low sensitivity for detecting stage I CRC and advanced adenomas (AA) (Sano et al., 2016) of annual FIT or gFOBT test has limited their effectiveness as screening tools for early stage CRC detection. On the other hand, despite the high accuracy of colonoscopy and flexible sigmoidoscopy, they all have shown low acceptance rate due to their bothersome bowel preparation, invasiveness and potential for complications (Niu et al., 2017).

*SEPT9* gene is a class of GTP-binding proteins (GTPases) involved in numerous cellular processes. It was demonstrated have multiple alternatively spliced transcripts encoding at least five characterized polypeptides designated v1–v5, ome of which have been associated with cervix, breast other cancer types (Wasserkort et al., 2013). The promoter region of the v2 transcript of *SEPT9* gene has been validated to be hypermethylated, which is highly specific to CRC carcinogenesis (Sun et al., 2018). Epi proColon 2.0 assay, a plasma-based *SEPT9* methylation test approved by CE, Chinese National Medical Products Administration (NMPA) and FDA (Lamb and Dhillon, 2017), showed 68.2–81.0% sensitivity and 78.2–98.9% specificity for CRC screening using 1/3 scoring algorithm (Johnson et al., 2014; Potter et al., 2014; Lamb and Dhillon, 2017). However, the sensitivities of *SEPT9* methylation for AA and CRC detection were relatively low, especially for early stage cancers (Church et al., 2014; Siegel et al., 2017; Zeng et al., 2018).

Several stool-based DNA methylation markers such as *SFRP2* (Wang and Tang, 2008), *NDRG4*, *BMP3* (Imperiale et al., 2014), and *SDC2* (Oh et al., 2017) have been previously described as potential markers for CRC screening. The multi-target stool DNA test approved by FDA in 2014 and recommended by ACS guideline is a combinatorial test for methylated *NDRG4* and *BMP3*, *KRAS* mutations and hemoglobin in stool samples. It has demonstrated 92.3 and 42.4% sensitivities, respectively, for detecting I–IV stage CRC and AA with a specificity of 86.7% (Lee et al., 2014). These observations suggested that stool DNA might be a better medium for CRC screening due to its direct origin from the gastrointestinal tract.

To evaluate the feasibility of stool methylated *SEPT9* test for CRC screening, we performed a methylated *SEPT9* test on plasma and stool specimens for a comparison of the two approaches of methylated *SEPT9* test in this study.

## Materials and Methods

### Sample Collection

**FIGURE 1:**
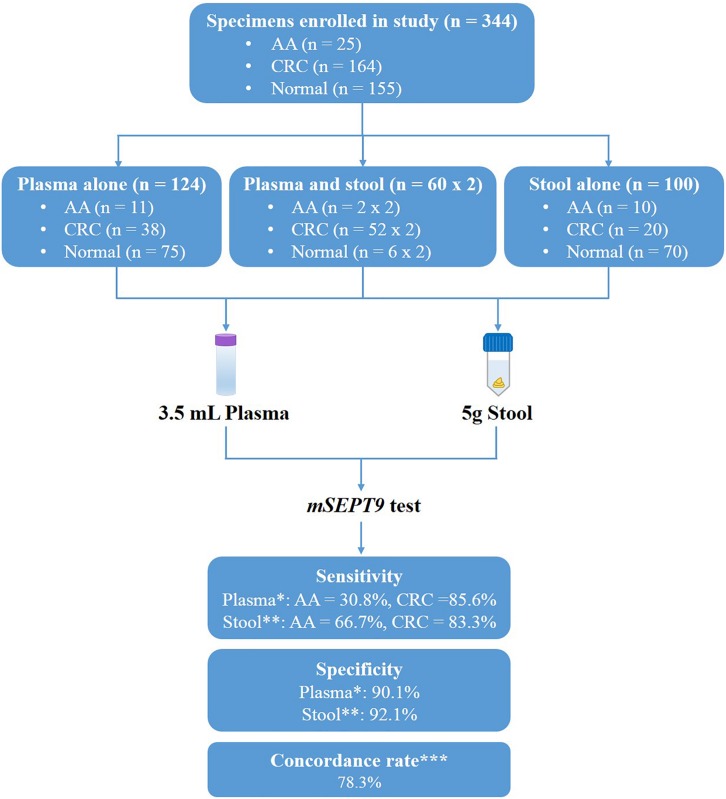
Study design and diagnostic accuracy measures obtained with methylated *SEPT9* test on plasma and stool samples. ^∗^Data analyzed by 1/3 algorithm. ^∗∗^Data analyzed by 3/3 algorithm with the mean Cp value of methylated *SEPT9* less than 40.0. ^∗∗∗^Concordance rate was calculated from the 42 paired plasma and stool samples.

There were 184 plasma samples and 160 stool samples in total, including 60 paired plasma and stool samples from 2 AA (adenomas measuring ≥1 cm in the greatest dimension, or with high-grade dysplasia or with ≥25% villous histologic features) patients, 52 CRC patients and six normal controls who were prospectively enrolled in the study from July 1, 2018 until December 31, 2019 (Figure 1). Plasma specimens were collected from 90 CRC patients, 13 AA patients, who underwent colonoscopy at the Affiliated Hospital of Xuzhou Medical University (Figure 1 and Table 1). Diagnoses of these patients were histologically confirmed by pathologists. Control plasma specimens were collected from 81 subjects without apparent digestive tract diseases (control individuals). Ten milliliter blood sample was drawn from each subject using a 10 mL K_3_EDTA tube (BD Biosciences) and stored at 4°C for up to 24 h. The plasma fractions were then separated and immediately frozen at −80°C until use.

**TABLE 1:** Characteristics of individuals examined by methylated *SEPT9* test.

Group	Characteristics		
	AA (*n* = 13)	Gender (%)	
		Male	38.5 (5)
		Female	61.5 (8)
		Age	
		Mean (min–max)	63 (49–90)
		Medium	60
Plasma	CRC (*n* = 90)	Gender (%)	
		Male	58.9 (53)
		Female	41.1 (37)
		Age	
		Mean (min–max)	61 (28–86)
		Medium	64
	Control (*n* = 81)	Gender (%)	
		Male	69.1 (56)
		Female	30.9 (25)
		Age	
		Mean (min–max)	42 (21–76)
		Medium	42
	AA (*n* = 12)	Gender (%)	
		Male	66.7 (8)
		Female	33.3 (4)
		Age	
		Mean (min–max)	59 (46–75)
		Medium	59
Stool	CRC (*n* = 72)	Gender (%)	
		Male	55.5 (40)
		Female	45.5 (32)
		Age	
		Mean (min–max)	60 (35–86)
		Medium	61
	Control (*n* = 76)	Gender (%)	
		Male	47.4 (36)
		Female	52.6 (40)
		Age	
		Mean (min–max)	45 (16–67)
		Medium	50

Stool samples from patients with histologically confirmed CRC (*n* = 72), AA (*n* = 12), and healthy normal subjects (*n* = 76) were obtained from the same hospital. All of the stool samples were collected prior to purgative bowel preparation or colonoscopy. Whole stools were collected in buckets mounted to the toilet seat, and then approximately 5 g of each stool specimen was transferred into a 50 mL tube which contained 25 mL of preservative buffer (Suzhou VersaBio Technologies Co. Ltd., Kunshan, China). All stool specimens were stored at −80°C before usage.

The details of all plasma and stool samples were shown in Table 1. The Institutional Review Board of the Affiliated Hospital of Xuzhou Medical University approved the study (Ethics Committee reference number: XYFY2018-KL081). All subjects provided written informed consent prior to participation.

### DNA Extraction, Bisulfite Treatment and Quantitative Real-Time PCR

For plasma samples, 3.5 mL plasma was separated form 10 mL blood and extracted using a cfDNA extraction kit (Suzhou VersaBio Technologies Co. Ltd., Kunshan, China). All stool samples were thawed for about 30 min at 15–30°C, and subsequently homogenized for 1 min with a shaker device. After homogenization, each stool sample was centrifuged for 20 min at 10,000*g*. One hundred and fifty microliter supernatants were removed for human genomic DNA extraction with a stool DNA extraction kit (Suzhou VersaBio Technologies Co. Ltd., Kunshan, China). Bisulfite conversion of purified plasma and stool DNA and purification of the converted products were performed with a bisulfite conversion kit (Suzhou VersaBio Technologies Co. Ltd.). All the kits were used according to the manufacturers’ instructions.

Purified DNA from the above steps was then tested by a methylated *SEPT9* test developed by Suzhou VersaBio Technologies Co. Ltd., which is a duplex methylated qPCR assay detecting promoter region of the *v2* transcript of *SEPT9* and an internal control (*ACTB*). Three PCR replicates were performed for each sample. Total reaction volume of qPCR was 30 μL including 15 μL PCR mastermix and 15 μL DNA. The qPCR experiments were performed on a LC480-II thermal cycler (Roche Diagnostics, Basel, Switzerland) following these cycling conditions: an initial activation at 95°C for 30 min, 50 cycles at 95°C for 10 s, then 56°C for 30 s, and a final cooling to 40°C for 30 s.

### Data Analysis

The result for a plasma specimen was considered “invalid” if its *ACTB* Cp value was greater than 36.0, and methylated *SEPT9* was considered “detected” if its Cp value was less than 45.0. The result for a stool sample was considered “invalid” if the Cp of *ACTB* was greater than 38.0, and methylated *SEPT9* was considered “detected” if its Cp value was less than 45.0.

Methylated *SEPT9* test is a qPCR reaction run in triplicates and therefore returns with several possible results depending on different algorithms (1/3, 2/3, or 3/3 for each target). According to this principle, the results of the methylated *SEPT9* test were analyzed with different algorithms to determine the optimal algorithm (Table 2).

**TABLE 2:** Positive detection rate (PDR) of plasma and stool methylated *SEPT9* tests for CRC with different algorithms.

All subjects		Number (N)	PDR in 1/3 [%(n/N)]	PDR in 2/3 [%(n/N)]	PDR in 3/3 [%(n/N)]	PDR in 3/3 and Mean Cp < 40 [%(n/N)]
	AA	13	30.8%(4/13)	7.7%(1/13)	7.7%(1/13)	7.7%(1/13)
	CRC	90	85.6%(77/90)	64.4%(58/90)	53.3%(48/90)	46.7%(42/90)
	I	18	77.8%(14/18)	33.3%(6/18)	16.7%(3/18)	16.7%(3/18)
Plasma	II	27	85.2%(23/27)	70.4%(19/27)	66.7%(18/27)	59.3%(16/27)
	III	26	92.3%(24/26)	69.2%(18/26)	50.0%(13/26)	34.6%(9/26)
	IV	10	80.0%(8/10)	70.0%(7/10)	60.0%(6/10)	60.0%(6/10)
	Unknown	9	88.9%(8/9)	88.9%(8/9)	88.9%(8/9)	88.9%(8/9)
	Normal	81	9.9%(8/81)	6.2(5/81)	3.7%(3/81)	1.2%(1/81)

	AA	12	83.3%(10/12)	75.0%(9/12)	66.7%(8/12)	66.7%(8/12)
	CRC	72	97.2%(70/72)	88.9%(64/72)	86.1%(62/72)	83.3%(60/72)
	I	15	100.0%(15/15)	93.3%(14/15)	86.7%(13/15)	86.7%(13/15)
Stool	II	18	94.4%(17/18)	94.4%(17/18)	94.4%(17/18)	94.4%(17/18)
	III	23	100.0%(23/23)	87.0%(20/23)	87.0%(20/23)	78.3%(18/23)
	IV	9	88.9%(8/9)	77.8%(7/9)	77.8%(7/9)	77.8%(7/9)
	Unknown	7	100.0%(7/7)	85.7%(6/7)	71.4%(5/7)	71.4%(5/7)
	Normal	76	51.3%(39/76)	30.3%(23/76)	13.1%(10/76)	7.9%(6/76)

*PDR in 1/3, 2/3, or 3/3 refer to detecting samples with 1/3, 2/3, or 3/3 algorithm.*

Statistical analysis was performed using IBM SPSS for Windows, Version 22.0, and *t*-test was used for the comparison between two samples at the significance level of *p* < 0.05. Receiver operating characteristic (ROC) curves were plotted using the mean Cp values from CRC and the Cp values from normal individuals. Because methylated *SEPT9* was not detected from most normal individuals by the qPCR reaction, we set the corresponding Cp values to 50.0 (the maximal number of PCR cycles) for such samples to plot the curve (Wu et al., 2016). To analyze the methylated *SEPT9* level, we also set a Cp value of 50.0 to the samples with no SEPT9 signal to calculate the mean Cp values of methylated *SEPT9*.

## Results

To compare the performance of methylated *SEPT9* test in plasma and stool specimens for CRC screening, 344 samples were collected from patients in the Affiliated Hospital of Xuzhou Medical University, including plasma samples only from 123 subjects, stool samples only from 100 subjects, and paired plasma and stool samples from 60 subjects (Figure 1). The details of age and gender information of all CRC, AA patients and normal controls were listed in Table 1.

As shown in Table 2, plasma methylated *SEPT9* test demonstrated relatively high sensitivity (85.6%) with a high specificity (90.1%) in detecting CRC with 1/3 algorithm [specificity equals to 1 minus positive detection rate (PDR) of normal subjects], but its sensitivity for detecting AA (30.8%) was low. With 2/3 or 3/3 algorithm, plasma methylated *SEPT9* test showed significant decrease in sensitivity for CRC and AA detection, especially for early stage CRC, while the specificities for CRC and AA detection were as high as 94.8 and 96.3%, respectively. On the contrary, stool methylated *SEPT9* test exhibited high sensitivities for detecting CRC (97.2%) and AA (83.3%) with 1/3 algorithm, but the specificity was only 48.7%. When 2/3 or 3/3 algorithm was used for analysis, stool methylated *SEPT9* test showed significant improvement in specificity (69.7 and 86.9%); however, although still relatively high, the sensitivities for detecting CRC (88.9 and 86.1%) and AA (75.0 and 66.7%) were reduced.

We further analyzed the stool methylated *SEPT9* test data using mean Cp values. The result revealed that if a cutoff value for the mean Cp value was set at 40.0, the specificity was improved to 92.1% without decreasing the sensitivities for detecting CRC and AA. The results of plasma methylated *SEPT9* test with 1/3 algorithm and stool methylated *SEPT9* test with 3/3 algorithm with a mean Cp value of less than 40.0 achieved the best balance between sensitivity and specificity in detecting both AA and CRC (Table 2). Therefore, all subsequent data were analyzed with these criteria.

**FIGURE 2:**
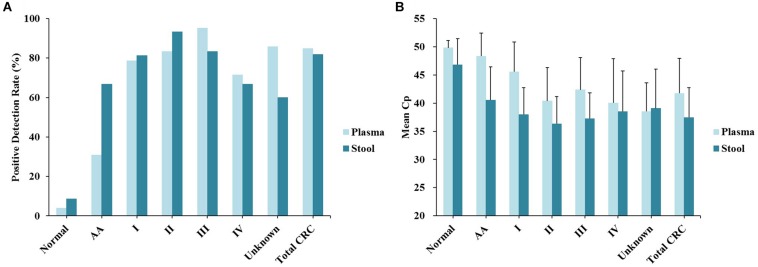
Sensitivity and specificity of plasma and stool methylated *SEPT9* tests in detecting AA and CRC across stages I–IV. **(A)** Positive detection rates for normal individuals, AA and all stages of CRC. **(B)** The mean Cp values of methylated *SEPT9* for normal individuals, AA and all stages of CRC.

**FIGURE 3:**
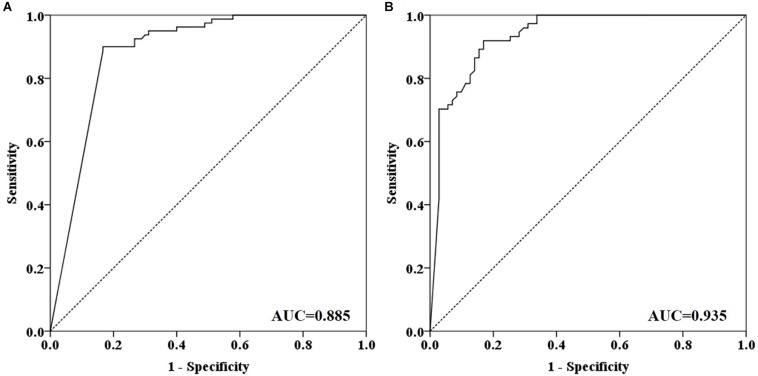
ROC curves for plasma and stool methylated *SEPT9* tests in detecting CRC. **(A)** ROC curves for plasma methylated *SEPT9* test. **(B)** ROC curves for stool methylated *SEPT9* test.

Out of 90 CRC and 8 AA plasma samples, methylated *SEPT9* was detected in 30.8% of AA (4/13), 77.8% of stage I (14/18), 85.2% of stage II (23/27), 92.3% of stage III (24/26), 80.0% of stage IV (8/10), and 88.9% of unknown stage (8/9) samples (Table 2 and Figure 2A). For 81 CRC and AA stool samples, methylated *SEPT9* was detected in 66.7% of AA (8/12), 86.7% of stage I (13/15), 94.4% of stage II (17/18), 78.3% of stage III (18/23), 77.8% of stage IV (7/9), and 71.4% of unknown stage (5/7) samples (Table 2 and Figure 2A). In order to compare the methylated *SEPT9* levels in plasma and stool samples, we calculated the mean Cp values of methylated *SEPT9* for normal individuals, AA and CRC. As showed in Figure 2B, the mean Cp values of methylated *SEPT9* of both plasma and stool samples of normal individuals were significantly higher than those of CRC samples, indicating that the methylated *SEPT9* levels of normal individuals were significantly lower than those of CRC patients (*p* < 0.001). Moreover, the methylated *SEPT9* levels in stool samples were significantly higher than that in plasma samples of AA and CRC patients (*p* < 0.001). ROC curves for methylated *SEPT9* test in detecting CRC with plasma and stool samples are shown in Figure 3. AUC for plasma methylated *SEPT9* in detecting CRC was 0.885 (95% CI: 0.832–0.938), and that for stool methylated *SEPT9* was 0.935 (95% CI: 0.895–0.975).

For the 60 plasma samples and their paired stool samples, plasma and stool methylated *SEPT9* tests showed the same PDR for CRC (86.5%) and normal (0.0%) subjects. Specifically, there were 39 stool-*SEPT9*+/plasma*-SEPT9*+ cases (all CRC patients), 8 stool-*SEPT9−*/plasma*-SEPT9−* cases (1 AA and 1 CRC cases and 6 normal individuals), 7 stool-*SEPT9*+/plasma*-SEPT9−* cases (1 AA and 6 CRC cases), and 6 stool-*SEPT9−*/plasma*-SEPT9*+ cases (6 CRC cases), demonstrating a concordance rate of 78.3% (Table 3). Furthermore, there was no significant difference among the PDRs of plasma methylated *SEPT9* test for different ages, genders, tumor locations or tumor sizes (*p* > 0.05, Table 4). The PDRs of stool methylated *SEPT9* test for different ages, genders and tumor sizes also showed no significant difference (*p* > 0.05, Table 4). However, significantly different PDRs by stool methylated *SEPT9* test were observed for different tumor locations (*p* < 0.05).

**TABLE 3:** The positive detection rates (PDR) and concordance rates for plasma and stool methylated *SEPT9* tests in detecting CRC and AA with paired plasma and stool samples.

Group	Number (N)	PDR in Plasma [%(n/N)]	PDR in Stool [%(n/N)]	Concordance rate [%(n/N)]
AA	2	0.0%(0/2)	50.0%(1/2)	50.0%(1/2)
CRC	52	86.5%(45/52)	86.5%(45/52)	76.9%(40/52)
I	11	81.8%(9/11)	81.8%(9/11)	63.6%(7/11)
II	16	87.5%(14/16)	93.8%(15/16)	87.5%(14/16)
III	13	84.6%(11/13)	76.9%(10/13)	76.9%(10/13)
IV	8	75.0%(6/8)	87.5%(7/8)	62.5%(5/8)
Unknown	4	100.0%(4/4)	75.0%(3/4)	75.0%(3/4)
Normal	6	0.0%(0/6)	0.0%(0/6)	100.0%(6/6)
All subjects	60	75.0%(45/60)	76.6%(46/60)	78.3%(47/60)

**TABLE 4:** Results of plasma and stool methylated *SEPT9* tests in detecting CRC for different ages, genders, tumor locations and tumor sizes.

	Plasma			Stool		
		
	Number (N)	PDR [%(n/N)]	*p*-value	Number (n/N)	PDR [%(n/N)]	*p*-value
**Age**						
<60	37	83.7% (31/37)	0.690	35	80.0% (28/35)	0.460
≥60	53	86.8% (46/53)		37	86.5% (32/37)	
**Gender**						
Male	53	90.6% (48/53)	0.106	39	94.8% (37/39)	0.020
Female	37	78.4% (29/37)		33	69.7% (23/33)	
**Location**						
Distal	39	87.2% (34/39)	0.814	33	72.7% (24/33)	0.056
Proximal	41	85.3% (35/41)		33	90.9% (30/33)	
N/A	10	80.0% (8/10)		6	100.0% (6/6)	
**Size**						
<3 cm	12	75.0% (9/12)	0.184^a^	11	90.9% (10/11)	0.549^a^
3–6 cm	48	89.6% (43/48)	0.618^b^	43	83.7% (36/43)	0.382^b^
>6 cm	13	84.6% (11/13)	0.548^c^	4	100.0% (4/4)	0.533^c^
N/A	17	82.4% (14/17)		14	78.6% (11/14)	

*N/A, not applicable. ^*a*^*p*-value between <3 cm and 3–6 cm; ^*b*^*p*-value between 3–6 cm, and >6 cm; ^*c*^*p*-value between <3 cm and >6 cm.*

## Discussion

Plasma methylated *SEPT9* is the only blood based biomarker approved by several countries for CRC screening, and its test has been used clinically for several years (Wu et al., 2016; Worm, 2018). However, the sensitivity of plasma *SEPT9* methylation for CRC detection was relatively low, especially for early stage cancers and AA (Siegel et al., 2017; Zeng et al., 2018). To improve the sensitivity of plasma methylated *SEPT9* test, several strategies have been proposed during the recent years. Among such strategies, the combination of multiple biomarkers and/or methods has become an effective approach in CRC diagnosis and screening to improve sensitivity (Song et al., 2017; Zhao et al., 2020). For example, it was reported that the sensitivities for CRC detection were 72.2 and 68.0%, respectively, for *SEPT9* methylation and FIT individually, but when test results for *SEPT9* methylation and FIT were combined, CRC detection rate increased to 88.7% (Johnson et al., 2014). Our earlier work on another blood based CRC screening assay, ColoDefense test that combines the detection of *SEPT9* and *SDC2* methylation in a single qPCR reaction, also improved the detection rates for early stage CRC and AA (Chen Y. et al., 2019; Zhao et al., 2019). To our knowledge, there has been only one published study examining the performance of methylated *SEPT9* in combination with other methylation biomarkers on stool samples, and the AUC of methylated *SEPT9* in that study was 0.815 (Chen J. et al., 2019). However, the performance of single methylated *SEPT9* test on stool samples for each CRC stage and the performance comparison between stool methylated *SEPT9* test and plasma methylated *SEPT9* test have never been reported.

As shown in Table 2, stool methylated *SEPT9* test had a low specificity in 1/3 algorithm probably because tumor DNA in stool had originated directly from gut (Glöckner et al., 2009), while the ctDNA from plasma should pass through various barriers of the body and degrades over time (Chen Y. et al., 2019). Meanwhile, Tóth et al. (2014) reported that methylated *SEPT9* could be detected in 100% (26/26) of adenomas and 97.1% (33/34) of CRC tissues, but methylated *SEPT9* was positive in only 30.8% (8/26) of adenomas and 88.2% (30/34) of CRC in plasma (), indicating a higher level of *SEPT9* methylation in tissues than that in plasma. Consistently, as stool DNA originated directly from AA and CRC tissues, the methylated *SEPT9* level in stool was higher than that in plasma. Therefore, the cut-off of stool methylated *SEPT9* test in this study was adjusted to 3/3 algorithm with the mean Cp value of methylated *SEPT9* less than 40.0. Based on this criterion, stool methylated *SEPT9* test achieved a similar sensitivity (*p* = 0.697) and specificity (*p* = 0.663) for CRC detection to those of plasma methylated *SEPT9* test, and the overall concordance rate was 78.3%. However, stool methylated *SEPT9* test showed 66.7 and 90.1% sensitivities for detecting AA and stage I–II CRC, 35.9 and 7.9% higher than those of plasma methylated *SEPT9* test (Table 2 and Figure 2). Therefore, stool methylated *SEPT9* test may be a better assay for AA and CRC screening than plasma methylated *SEPT9* test.

During the past decade, many methylated biomarkers related to CRC have been reported (Luo et al., 2014; Patai et al., 2015), and several plasma methylated DNA tests for CRC screening have been developed in addition to methylated *SEPT9* test. Zhang et al. (2015) showed that combined detection of plasma methylated *GATA5* and *SFRP2* could detect 63.3% adenomas and 73.7% CRC with a specificity of 66.0%. Barták et al. (2017) reported a biomarker panel containing methylated *SFRP1*, *SFRP2*, *SDC2*, and *PRIMA1*, that could distinguish CRC with 91.5% sensitivity and 97.3% specificity and AA with 89.2% sensitivity and 86.5% specificity from controls (Barták et al., 2017), showing higher sensitivities for CRC and adenoma detection than those of plasma *SEPT9* test (Church et al., 2014; Worm, 2018). The assay of Barták et al. (2017) was based on a nested PCR approach to improve sensitivity, especially for adenomas, but the methylated *SEPT9* tests in our study and previous publications used one-step qMSP assays (Potter et al., 2014; Lamb and Dhillon, 2017), which were much easier to perform and less prone to cross-contamination in clinical application. Despite this limitation, the studies reported by Zhang et al. (2015) and Barták et al. (2017) indicated that combining methylated *SEPT9* with other methylation biomarkers, such as methylated *SFRP2*, could to improve the sensitivity of detecting AA in plasma samples.

Stool DNA has also been applied for CRC screening for several years. In 2014, FDA approved a multi-target stool DNA test, Cologuard, which combines 2 DNA methylation biomarkers, 7 *KRAS* mutation sites as well as an immunochemical assay for human hemoglobin. It detected 92.3% of CRC and 42.4% of AA with a specificity of 86.7% (Imperiale et al., 2014). However, its high price and cumbersome procedure (Niu et al., 2017) made it unsuitable for developing countries like China. In 2018, Chinese NMPA also approved a stool methylated DNA test, which detects methylated *SDC2* and *ACTB* (a reference gene for human DNA quantity) by a duplex qPCR assay. This assay detected 81.1% of CRC and 58.2% of AA at a specificity of 93.3% (Niu et al., 2017). Due to its high sensitivities for detecting early stage CRC (89.7% detection rate for stage I CRC) and AA, Cologuard was incorporated in the updated CRC screening guideline from ACS in 2018 (Worm, 2018). On the contrary, the new ACS guideline did not recommend the plasma methylated *SEPT9* test, Epi proColon 2.0, for CRC screening due to its low sensitivities for detecting early stage CRC and AA (Worm, 2018). In this study, we showed that stool methylated *SEPT9* test had high sensitivities (66.7 and 86.7%) in detecting AA and stage I CRC (Table 2), suggesting that stool methylated *SEPT9* test could identify more AA patients than Cologuard and methylated *SDC2* test (Lee et al., 2014;Niu et al., 2017), and its performance in detecting stage I CRC was comparable to that of Cologuard (Imperiale et al., 2014). Moreover, stool methylated *SEPT9* test was a single-tube duplex qPCR assay. In comparison, Cologuard detects 2 DNA methylation biomarkers, 7 *KRAS* mutation sites and human hemoglobin in several reactions for a single sample, thus increasing the operational cost and complexity. As only one plasma methylated *SEPT9* test has been approved by FDA (Wu et al., 2016), the stool methylated *SEPT9* test examined in this study provided another lower-cost assay with comparable performance to Cologuard that has the potential to receive international regulatory approval for CRC screening and prevention, especially for developing countries.

However, there were several limitations in this study. For example, the number of AA samples examined in this study was relatively low. In previous studies, Tóth et al. (2014) enrolled 26 adenomas plasma samples for evaluating plasma methylated *SEPT9* test, Imperiale et al. (2014) collected 757 stool samples to evaluate the performance of Cologuard, and Niu et al. (2017) analyzed performance of methylated *SDC2* in 122 adenoma stool samples. In this study, only 13 AA plasma samples and 12 AA stool samples were collected, thus further increasing the number of enrolled AA patients would make it possible to distinguish the diagnostic performance between plasma methylated *SEPT9* test and stool methylated *SEPT9* test for AA detection. Moreover, as Toth et al. (2012); Tóth et al. (2017)) reported that the circadian rhythm of DNA amount in plasma and the sidedness of tumor might affect the performance of plasma methylated *SEPT9* test, it would be worthwhile to examine the circadian rhythm of methylated *SEPT9* in stool and plasma samples and to analyze the effect of sidedness on CRC detection in future studies.

## Conclusion

In this study, we evaluated the feasibility of stool methylated *SEPT9* test for CRC screening and compared the performance of methylated *SEPT9* test on stool and plasma specimens. The results demonstrated that stool methylated *SEPT9* test and plasma methylated *SEPT9* test had similar sensitivity and specificity for all stage CRC detection, but stool methylated *SEPT9* test exhibited higher sensitivities for detecting AA and early stage CRC, suggesting that stool methylated *SEPT9* test may be an viable alternative for CRC screening with high sensitivity and specificity.

## Data Availability Statement

The datasets used and/or analyzed during the current study are available from the corresponding author on reasonable request.

## Ethics Statement

The studies involving human participants were reviewed and approved by the Institutional Review Board of the Affiliated Hospital of Xuzhou Medical University (Ethics Committee reference number: XYFY2018-KL081). The patients/participants provided their written informed consent to participate in this study.

## Author Contributions

GZ, YL, HL, and YM performed the statistical analyses and drafted the manuscript. YL, JM, HL, XL, SL, YZ, SX, MZ, and SF participated in sample collection and the data analysis. GZ, YL, SX, MZ, and SF conceived of the study and participated in the design, and coordination of the study. All authors read and approved the final manuscript.

## Conflict of Interest

GZ and SX are employees of Suzhou VersaBio Technologies Co., Ltd. SX is the shareholder of Suzhou VersaBio Technologies Co., Ltd. The remaining authors declare that the research was conducted in the absence of any commercial or financial relationships that could be construed as a potential conflict of interest.
